# Cinnarizinium bis­(*p*-toluene­sulfonate) dihydrate

**DOI:** 10.1107/S1600536813003991

**Published:** 2013-03-06

**Authors:** C. N. Kavitha, Ray J. Butcher, Jerry P. Jasinski, H. S. Yathirajan, A. S. Dayananda

**Affiliations:** a Department of Studies in Chemistry, University of Mysore, Manasagangotri, Mysore 570 006, India; b Department of Chemistry, Howard University, 525 College Street NW, Washington, DC 20059, USA; c Department of Chemistry, Keene State College, 229 Main Street, Keene, NH 03435-2001, USA

## Abstract

The asymmetric unit of the title salt [systematic name: 1-benzhydryl-4-cinnamylpiperazine-1,4-diium bis­(*p*-toluene­sulfonate) dihydrate], C_26_H_30_N_2_^2+^·2C_7_H_7_O_3_S^−^·2H_2_O, consists of a diprotonated cinnarizinium cation hydrogen bonded through two water mol­ecules to two independent *p*-toluene­sulfonate anions, one which is disordered over two sets of sites in a 0.793 (3):0.207 (3) ratio. In the cation, the piperazine ring adopts a chair configuration and contains two positively charged N atoms with quarternery character. The dihedral angle between the two benzene rings in the benzhydr­yl group is 71.8 (1)°. The benzene ring flanked opposite the piperazine ring is twisted by 75.9 (9) and 8.8 (3)° from these two benzene rings. In the crystal, the [N—H⋯O_water_—H⋯O( S)]_2_ hydrogen-bonded asymmetric unit is connected by further O—H⋯O hydrogen bonds linking the components into chains along [100].

## Related literature

For cinnarizine (systematic name: 1-benzhydryl-4-cinnamyl-piperazine) as a nootropic drug, see: Towse (1980 ▶). For cinnarizine in allergic disorders, see: Barrett & Zolov (1960 ▶). For related structures, see: Bertolasi *et al.* (1980 ▶); Dayananda *et al.* (2012 ▶); Jasinski *et al.* (2011 ▶); Mouillé *et al.* (1975 ▶); Smith *et al.* (2001 ▶); Song *et al.* (2012 ▶). For puckering parameters, see: Cremer & Pople (1975 ▶). For standard bond lengths, see: Allen *et al.* (1987 ▶).
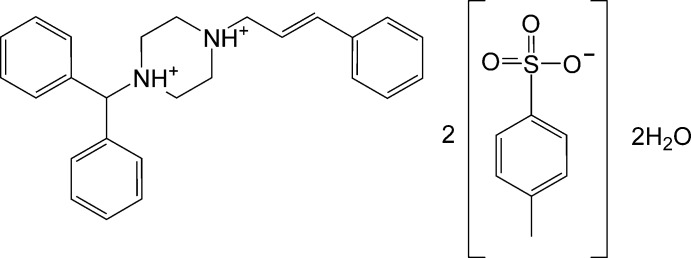


## Experimental

### 

#### Crystal data

C_26_H_30_N_2_^2+^·2C_7_H_7_O_3_S^−^·2H_2_O
*M* *_r_* = 748.92
Monoclinic, 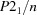

*a* = 10.0845 (2) Å
*b* = 14.6026 (3) Å
*c* = 25.8591 (6) Å
β = 93.414 (2)°
*V* = 3801.25 (14) Å^3^
*Z* = 4
Cu *K*α radiation
μ = 1.72 mm^−1^
*T* = 100 K
0.47 × 0.28 × 0.17 mm

#### Data collection

Agilent Xcalibur Ruby Gemini diffractometer
Absorption correction: multi-scan (*CrysAlis PRO* and *CrysAlis RED*; Agilent, 2012 ▶) *T*_min_ = 0.705, *T*_max_ = 1.000
14591 measured reflections
7666 independent reflections
7051 reflections with *I* > 2σ(*I*)
*R*_int_ = 0.033

#### Refinement

*R*[*F* ^2^ > 2σ(*F* ^2^)] = 0.054
*wR*(*F* ^2^) = 0.140
*S* = 1.06
7666 reflections
541 parameters
H atoms treated by a mixture of independent and constrained refinement
Δρ_max_ = 0.60 e Å^−3^
Δρ_min_ = −0.37 e Å^−3^

### 

Data collection: *CrysAlis PRO* (Agilent, 2012 ▶); cell refinement: *CrysAlis PRO*; data reduction: *CrysAlis PRO*; program(s) used to solve structure: *SHELXS97* (Sheldrick, 2008 ▶); program(s) used to refine structure: *SHELXL97* (Sheldrick, 2008 ▶); molecular graphics: *SHELXTL* (Sheldrick, 2008 ▶); software used to prepare material for publication: *SHELXTL*.

## Supplementary Material

Crystal structure: contains datablock(s) global, I. DOI: 10.1107/S1600536813003991/lh5578sup1.cif

Structure factors: contains datablock(s) I. DOI: 10.1107/S1600536813003991/lh5578Isup2.hkl

Supplementary material file. DOI: 10.1107/S1600536813003991/lh5578Isup3.cml

Additional supplementary materials:  crystallographic information; 3D view; checkCIF report

## Figures and Tables

**Table 1:** Hydrogen-bond geometry (Å, °)

*D*—H⋯*A*	*D*—H	H⋯*A*	*D*⋯*A*	*D*—H⋯*A*
N1—H1*N*⋯O1*W*	0.84 (3)	1.86 (3)	2.699 (2)	174 (3)
N2—H2*N*⋯O2*W*	0.98 (3)	1.69 (3)	2.661 (3)	173 (3)
O1*W*—H1*W*1⋯O1*B* ^i^	0.84 (3)	1.86 (3)	2.685 (2)	167 (3)
O1*W*—H1*W*1⋯O3*C* ^i^	0.84 (3)	2.07 (3)	2.826 (9)	149 (3)
O1*W*—H1*W*2⋯O3*A*	0.91 (4)	1.85 (4)	2.747 (2)	168 (4)
O2*W*—H2*W*1⋯O2*A* ^ii^	0.86 (4)	1.88 (4)	2.739 (3)	176 (3)
O2*W*—H2*W*2⋯O1*C*	0.83 (5)	1.59 (5)	2.336 (10)	147 (4)
O2*W*—H2*W*2⋯O1*B*	0.83 (5)	2.01 (5)	2.830 (3)	169 (4)

Symmetry codes: (i) 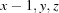; (ii) 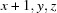.

**Fig. 1.**
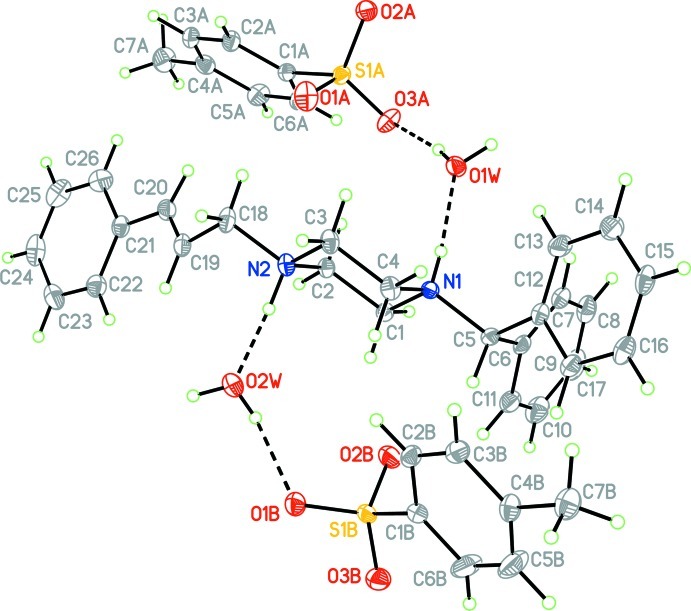
Molecular structure of the title compound showing 30% probability displacement ellipsoids. Dashed lines indicate N—H···O and O—H···O hydrogen bonds. One of the two independent p-toluenesulfonate anions is disordered in a 0.793 (3):0.207 (3) ratio and only the major component (B) is displayed.

**Fig. 2.**
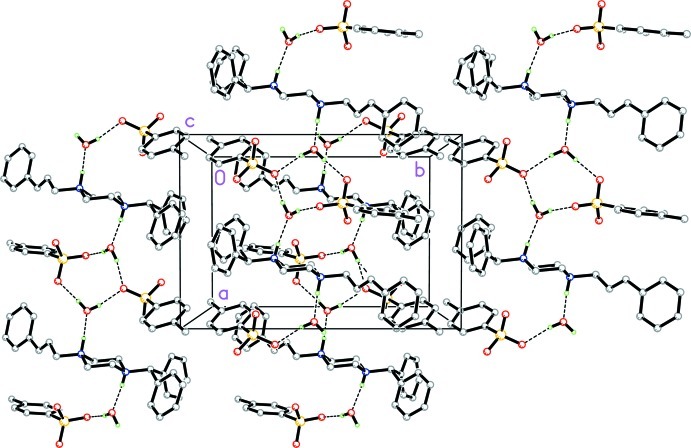
Packing diagram of the title compound viewed along the c axis. Dashed lines indicate N—H···O, and O—H···O hydrogen bonds. H atoms not involved in hydrogen bonding have been removed for clarity.

## supplementary crystallographic information

### Comment

Cinnarizine (Iupac name: 1-benzhydryl-4-cinnamyl-piperazine) is an antihistamine which is mainly used for the control of nausea and vomiting due to motion sickness. Cinnarizine could be also viewed as a nootropic drug because of its vasorelaxating abilities (due to calcium channel blockage), which happen mostly in the brain and is also used as a labyrinthine sedative (Towse, 1980). A clinical evaluation of cinnarizine in various allergic disorders is published (Barrett & Zolov, 1960). Cinnarizine can be used in scuba divers without an increased risk of central nervous system oxygen toxicity. The crystal structures of some related compounds viz., cinnarizine (Mouillé *et al.*, 1975), cyclizine hydrochloride (Bertolasi *et al.*, 1980), cinnarizinium dipicrate (Jasinski *et al.*, 2011), cinnarizinium 3,5-dinitrosalicylate (Dayananda *et al.*, 2012), cinnarizinium picrate (Song *et al.*, 2012), have been reported. In view of the importance of cinnarizine, this paper reports the crystal structure of the title compound, C_26_H_30_N_2+_ . C_14_H_14_O_6_S_2_^-^ . 2H_2_O, (I).

The asymmetric unit of the title salt (Fig .1), C_26_H_30_N_2+_ . C_14_H_14_O_6_S_2_^-^ . 2H_2_O consists of a diprotonated cinnarizinium cation hydrogen bonded through two water molecules to two independent *p*-toluenesulfonate anions, one of which is disordered in a 0.793 (3):0.207 (3) ratio. In the cation, the piperazine ring, N1/C1/C2/N2/C3/C4, adopts a chair configuration with puckering parameters Q = 0.585 (2)Å, θ = 180.0 (19)°, φ = 150 (10)° (Cremer & Pople, 1975) and contains two positively charged N atoms with quarternery character. The dihedral angle between the two benzene rings in the benzhydryl group is 71.8 (1)°. The benzene ring flanked opposite the piperazine ring is twisted by 75.9 (9)° and 8.8 (3)° from these two benzene rings. The bond lengths in the title compound are as expected (Allen, *et al.*, 1987).

In the crystal, N—H···O hydrogen bonds (Table 1) from the diprotonated N atoms of the cation to nearby water molecules which subsequentially form O—H···O hydrogen bonds to the two *p*-toluenesulfonate anions link the components into infinite 1-D chains along [100] (Fig. 2).

### Experimental

Cinnarizine (3.68 g, 0.01 mol) and *p*-toluenesuphonic acid monohydrate (1.90 g, 0.01 mol) were dissolved in hot dimethyl sulphoxide solution and stirred over a heating magnetic stirrer for few minutes. The resulting solution was allowed to cool slowly at room temperature. X-ray quality crystals of the title compound appeared after a few days. (M.P.: 373–378 K).

### Refinement

HN1, HN2, H1W1, H1W2, H2W1 and H2W2 were located by Fourier maps and refined isotropically. All of the remaining H atoms were placed in their calculated positions and then refined using the riding model with Atom—H lengths of 0.95Å (CH), 0.99Å (CH_2_) and 0.98Å (CH_3_) Isotropic displacement parameters for these atoms were set to 1.19-1.21 (CH, CH_2_) or 1.49-1.51 (CH_3_) times *U*_eq_ of the parent atom.

### Crystal data

**Table:**

C_26_H_30_N_2_^2+^·2C_7_H_7_O_3_S^−^·2H_2_O	*F*(000) = 1592
*M**_r_* = 748.92	*D*_x_ = 1.309 Mg m^−^^3^
Monoclinic, *P*2_1_/*n*	Cu *K*α radiation, λ = 1.54184 Å
Hall symbol: -P 2yn	Cell parameters from 7892 reflections
*a* = 10.0845 (2) Å	θ = 3.0–75.5°
*b* = 14.6026 (3) Å	µ = 1.72 mm^−^^1^
*c* = 25.8591 (6) Å	*T* = 100 K
β = 93.414 (2)°	Triangular prism, colorless
*V* = 3801.25 (14) Å^3^	0.47 × 0.28 × 0.17 mm
*Z* = 4	

### Data collection

**Table:**

Agilent Xcalibur Ruby Gemini diffractometer	7666 independent reflections
Radiation source: Enhance (Cu) X-ray Source	7051 reflections with *I* > 2σ(*I*)
Graphite monochromator	*R*_int_ = 0.033
Detector resolution: 10.5081 pixels mm^-1^	θ_max_ = 75.7°, θ_min_ = 3.4°
ω scans	*h* = −12→9
Absorption correction: multi-scan (*CrysAlis PRO* and *CrysAlis RED*; Agilent, 2012)	*k* = −12→18
*T*_min_ = 0.705, *T*_max_ = 1.000	*l* = −32→31
14591 measured reflections	

### Refinement

**Table:**

Refinement on *F*^2^	Primary atom site location: structure-invariant direct methods
Least-squares matrix: full	Secondary atom site location: difference Fourier map
*R*[*F*^2^ > 2σ(*F*^2^)] = 0.054	Hydrogen site location: inferred from neighbouring sites
*wR*(*F*^2^) = 0.140	H atoms treated by a mixture of independent and constrained refinement
*S* = 1.06	*w* = 1/[σ^2^(*F*_o_^2^) + (0.0664*P*)^2^ + 2.4545*P*] where *P* = (*F*_o_^2^ + 2*F*_c_^2^)/3
7666 reflections	(Δ/σ)_max_ = 0.001
541 parameters	Δρ_max_ = 0.60 e Å^−^^3^
0 restraints	Δρ_min_ = −0.37 e Å^−^^3^

### Special details

**Table:**

Geometry. All esds (except the esd in the dihedral angle between two l.s. planes) are estimated using the full covariance matrix. The cell esds are taken into account individually in the estimation of esds in distances, angles and torsion angles; correlations between esds in cell parameters are only used when they are defined by crystal symmetry. An approximate (isotropic) treatment of cell esds is used for estimating esds involving l.s. planes.
Refinement. Refinement of *F*^2^ against ALL reflections. The weighted *R*-factor *wR* and goodness of fit *S* are based on *F*^2^, conventional *R*-factors *R* are based on *F*, with *F* set to zero for negative *F*^2^. The threshold expression of *F*^2^ > σ(*F*^2^) is used only for calculating *R*-factors(gt) *etc*. and is not relevant to the choice of reflections for refinement. *R*-factors based on *F*^2^ are statistically about twice as large as those based on *F*, and *R*- factors based on ALL data will be even larger.

### Fractional atomic coordinates and isotropic or equivalent isotropic displacement parameters (Å^2^)

**Table:**

	*x*	*y*	*z*	*U*_iso_*/*U*_eq_	Occ. (<1)
N1	0.65374 (16)	0.31271 (11)	0.56450 (6)	0.0214 (3)	
N2	0.76219 (17)	0.49893 (11)	0.56859 (6)	0.0266 (3)	
C1	0.72326 (19)	0.35115 (13)	0.61278 (7)	0.0242 (4)	
H1A	0.8187	0.3349	0.6136	0.029*	
H1B	0.6851	0.3237	0.6436	0.029*	
C2	0.70870 (19)	0.45477 (13)	0.61469 (7)	0.0240 (4)	
H2A	0.6136	0.4707	0.6163	0.029*	
H2B	0.7566	0.4785	0.6464	0.029*	
C3	0.6939 (2)	0.46042 (14)	0.52066 (7)	0.0288 (4)	
H3A	0.7316	0.4884	0.4899	0.035*	
H3B	0.5983	0.4759	0.5199	0.035*	
C4	0.7099 (2)	0.35766 (14)	0.51847 (7)	0.0287 (4)	
H4A	0.6639	0.3340	0.4863	0.034*	
H4B	0.8054	0.3423	0.5176	0.034*	
C5	0.67147 (19)	0.20872 (13)	0.56135 (8)	0.0253 (4)	
H5A	0.7685	0.1970	0.5586	0.030*	
C6	0.6320 (2)	0.16318 (13)	0.61093 (8)	0.0265 (4)	
C7	0.5008 (2)	0.14981 (14)	0.62210 (9)	0.0327 (4)	
H7A	0.4311	0.1701	0.5985	0.039*	
C8	0.4715 (3)	0.10679 (17)	0.66771 (10)	0.0445 (6)	
H8A	0.3815	0.0978	0.6754	0.053*	
C9	0.5728 (3)	0.07656 (17)	0.70246 (10)	0.0502 (7)	
H9A	0.5521	0.0469	0.7337	0.060*	
C10	0.7033 (3)	0.08991 (18)	0.69133 (10)	0.0491 (6)	
H10A	0.7728	0.0693	0.7149	0.059*	
C11	0.7335 (2)	0.13331 (15)	0.64591 (9)	0.0370 (5)	
H11A	0.8236	0.1428	0.6385	0.044*	
C12	0.6004 (2)	0.16984 (13)	0.51271 (7)	0.0260 (4)	
C13	0.4735 (2)	0.19524 (16)	0.49385 (9)	0.0391 (5)	
H13A	0.4276	0.2424	0.5107	0.047*	
C14	0.4132 (3)	0.15240 (17)	0.45060 (10)	0.0422 (5)	
H14A	0.3269	0.1706	0.4379	0.051*	
C15	0.4790 (3)	0.08314 (15)	0.42614 (8)	0.0372 (5)	
H15A	0.4376	0.0530	0.3969	0.045*	
C16	0.6051 (3)	0.05796 (15)	0.44440 (9)	0.0385 (5)	
H16A	0.6504	0.0104	0.4277	0.046*	
C17	0.6659 (2)	0.10176 (14)	0.48701 (8)	0.0315 (4)	
H17A	0.7536	0.0849	0.4988	0.038*	
C18	0.7481 (2)	0.60194 (14)	0.56963 (8)	0.0338 (5)	
H18A	0.7788	0.6277	0.5370	0.041*	
H18B	0.6532	0.6180	0.5717	0.041*	
C19	0.8262 (2)	0.64383 (14)	0.61442 (8)	0.0312 (4)	
H19A	0.9187	0.6317	0.6187	0.037*	
C20	0.7705 (2)	0.69750 (15)	0.64845 (8)	0.0315 (4)	
H20A	0.6764	0.7021	0.6456	0.038*	
C21	0.8404 (2)	0.75044 (14)	0.69008 (8)	0.0305 (4)	
C22	0.9735 (2)	0.73627 (16)	0.70559 (9)	0.0360 (5)	
H22A	1.0225	0.6896	0.6896	0.043*	
C23	1.0346 (3)	0.78994 (19)	0.74422 (10)	0.0438 (6)	
H23A	1.1251	0.7793	0.7549	0.053*	
C24	0.9647 (3)	0.85916 (18)	0.76743 (9)	0.0474 (6)	
H24A	1.0071	0.8964	0.7936	0.057*	
C25	0.8337 (3)	0.87308 (18)	0.75206 (9)	0.0487 (6)	
H25A	0.7854	0.9203	0.7678	0.058*	
C26	0.7715 (3)	0.81953 (18)	0.71422 (9)	0.0405 (5)	
H26A	0.6804	0.8297	0.7044	0.049*	
S1B	1.10167 (7)	0.22582 (6)	0.55532 (5)	0.0278 (2)	0.793 (3)
O1B	1.16096 (19)	0.31402 (13)	0.54245 (8)	0.0344 (5)	0.793 (3)
O2B	0.9784 (3)	0.2394 (3)	0.58013 (13)	0.0404 (7)	0.793 (3)
O3B	1.1951 (2)	0.16581 (15)	0.58372 (8)	0.0395 (5)	0.793 (3)
C1B	1.0592 (4)	0.1723 (3)	0.4955 (2)	0.0299 (9)	0.793 (3)
C2B	0.9819 (4)	0.2188 (2)	0.45755 (16)	0.0297 (7)	0.793 (3)
H2BA	0.9573	0.2808	0.4629	0.036*	0.793 (3)
C3B	0.9407 (4)	0.1743 (3)	0.41185 (16)	0.0316 (8)	0.793 (3)
H3BA	0.8864	0.2056	0.3863	0.038*	0.793 (3)
C4B	0.9788 (5)	0.0836 (3)	0.4034 (2)	0.0344 (10)	0.793 (3)
C5B	1.0551 (5)	0.0398 (3)	0.44126 (19)	0.0583 (12)	0.793 (3)
H5BA	1.0808	−0.0220	0.4361	0.070*	0.793 (3)
C6B	1.0959 (5)	0.0837 (3)	0.48724 (19)	0.0547 (12)	0.793 (3)
H6BA	1.1494	0.0521	0.5129	0.066*	0.793 (3)
C7B	0.9309 (5)	0.0331 (3)	0.35407 (16)	0.0435 (9)	0.793 (3)
H7BA	0.9093	−0.0303	0.3626	0.065*	0.793 (3)
H7BB	1.0010	0.0338	0.3294	0.065*	0.793 (3)
H7BC	0.8515	0.0635	0.3386	0.065*	0.793 (3)
S1C	1.0840 (4)	0.2469 (3)	0.53724 (19)	0.0396 (10)*	0.207 (3)
O1C	1.0619 (9)	0.3411 (7)	0.5240 (4)	0.049 (2)*	0.207 (3)
O2C	0.9917 (16)	0.2132 (10)	0.5773 (6)	0.045 (4)*	0.207 (3)
O3C	1.2223 (8)	0.2279 (6)	0.5563 (3)	0.040 (2)*	0.207 (3)
C1C	1.0485 (19)	0.1819 (12)	0.4812 (6)	0.021 (4)*	0.207 (3)
C2C	0.9489 (14)	0.2030 (10)	0.4471 (7)	0.025 (4)*	0.207 (3)
H2CA	0.8965	0.2557	0.4529	0.030*	0.207 (3)
C3C	0.9204 (18)	0.1512 (12)	0.4045 (7)	0.030 (5)*	0.207 (3)
H3CA	0.8484	0.1708	0.3818	0.036*	0.207 (3)
C4C	0.985 (2)	0.0736 (14)	0.3910 (7)	0.027 (5)*	0.207 (3)
C5C	1.1030 (12)	0.0533 (9)	0.4276 (5)	0.027 (3)*	0.207 (3)
H5CA	1.1600	0.0039	0.4199	0.033*	0.207 (3)
C6C	1.1315 (13)	0.1042 (10)	0.4720 (5)	0.031 (3)*	0.207 (3)
H6CA	1.2034	0.0883	0.4957	0.037*	0.207 (3)
C7C	0.9595 (19)	0.0133 (13)	0.3475 (8)	0.040 (5)*	0.207 (3)
H7CA	1.0430	−0.0145	0.3380	0.060*	0.207 (3)
H7CB	0.9202	0.0483	0.3181	0.060*	0.207 (3)
H7CC	0.8977	−0.0349	0.3569	0.060*	0.207 (3)
S1A	0.33646 (5)	0.58716 (3)	0.629055 (18)	0.02690 (13)	
O1A	0.43410 (19)	0.60649 (13)	0.59122 (6)	0.0434 (4)	
O2A	0.20127 (17)	0.60346 (11)	0.60795 (7)	0.0444 (4)	
O3A	0.35469 (18)	0.49646 (10)	0.65172 (6)	0.0378 (4)	
C1A	0.36629 (19)	0.66653 (14)	0.68031 (8)	0.0263 (4)	
C2A	0.3782 (2)	0.75874 (15)	0.66805 (8)	0.0310 (4)	
H2AA	0.3725	0.7778	0.6329	0.037*	
C3A	0.3983 (2)	0.82273 (15)	0.70733 (9)	0.0342 (5)	
H3AA	0.4051	0.8858	0.6988	0.041*	
C4A	0.4086 (2)	0.79587 (16)	0.75925 (9)	0.0345 (5)	
C5A	0.3980 (2)	0.70304 (17)	0.77068 (8)	0.0385 (5)	
H5AA	0.4057	0.6836	0.8058	0.046*	
C6A	0.3765 (2)	0.63828 (15)	0.73161 (8)	0.0349 (5)	
H6AA	0.3689	0.5752	0.7400	0.042*	
C7A	0.4288 (3)	0.86573 (19)	0.80187 (10)	0.0494 (6)	
H7AA	0.4444	0.8343	0.8352	0.074*	
H7AB	0.3495	0.9042	0.8029	0.074*	
H7AC	0.5058	0.9041	0.7953	0.074*	
O1W	0.40391 (15)	0.37324 (11)	0.57489 (7)	0.0343 (3)	
O2W	1.02377 (19)	0.47283 (15)	0.57219 (10)	0.0560 (5)	
H1N	0.574 (3)	0.3282 (18)	0.5665 (9)	0.027 (6)*	
H2N	0.857 (3)	0.487 (2)	0.5673 (11)	0.039 (7)*	
H1W1	0.330 (3)	0.348 (2)	0.5678 (11)	0.042 (8)*	
H1W2	0.379 (4)	0.409 (3)	0.6016 (15)	0.070 (11)*	
H2W1	1.082 (3)	0.512 (3)	0.5843 (13)	0.057 (9)*	
H2W2	1.056 (4)	0.422 (3)	0.5652 (16)	0.081 (13)*	

### Atomic displacement parameters (Å^2^)

**Table:**

	*U*^11^	*U*^22^	*U*^33^	*U*^12^	*U*^13^	*U*^23^
N1	0.0199 (7)	0.0216 (7)	0.0226 (7)	0.0008 (6)	0.0003 (6)	−0.0043 (6)
N2	0.0293 (9)	0.0253 (8)	0.0249 (8)	−0.0070 (7)	0.0004 (6)	−0.0010 (6)
C1	0.0274 (9)	0.0227 (9)	0.0221 (8)	−0.0004 (7)	−0.0019 (7)	−0.0025 (7)
C2	0.0262 (9)	0.0232 (9)	0.0228 (8)	−0.0033 (7)	0.0022 (7)	−0.0021 (7)
C3	0.0340 (10)	0.0298 (10)	0.0221 (9)	−0.0090 (8)	−0.0013 (7)	0.0005 (7)
C4	0.0329 (10)	0.0314 (10)	0.0219 (9)	−0.0042 (8)	0.0029 (7)	−0.0038 (7)
C5	0.0235 (9)	0.0221 (9)	0.0302 (9)	0.0035 (7)	0.0005 (7)	−0.0051 (7)
C6	0.0327 (10)	0.0179 (8)	0.0288 (9)	0.0018 (7)	0.0002 (8)	−0.0056 (7)
C7	0.0369 (11)	0.0230 (9)	0.0383 (11)	0.0017 (8)	0.0024 (9)	−0.0066 (8)
C8	0.0534 (14)	0.0318 (11)	0.0501 (14)	−0.0093 (10)	0.0187 (11)	−0.0117 (10)
C9	0.086 (2)	0.0338 (12)	0.0317 (11)	−0.0092 (13)	0.0073 (12)	0.0006 (9)
C10	0.0692 (18)	0.0359 (12)	0.0404 (13)	0.0005 (12)	−0.0119 (12)	0.0078 (10)
C11	0.0433 (12)	0.0283 (10)	0.0384 (11)	0.0027 (9)	−0.0053 (9)	0.0008 (9)
C12	0.0320 (10)	0.0203 (8)	0.0258 (9)	−0.0018 (7)	0.0015 (7)	−0.0024 (7)
C13	0.0416 (12)	0.0324 (11)	0.0421 (12)	0.0089 (9)	−0.0077 (10)	−0.0143 (9)
C14	0.0473 (13)	0.0342 (12)	0.0430 (12)	0.0018 (10)	−0.0160 (10)	−0.0033 (10)
C15	0.0593 (14)	0.0248 (10)	0.0265 (10)	−0.0090 (9)	−0.0065 (9)	−0.0032 (8)
C16	0.0542 (14)	0.0263 (10)	0.0357 (11)	−0.0004 (10)	0.0067 (10)	−0.0095 (9)
C17	0.0357 (11)	0.0236 (9)	0.0353 (10)	0.0020 (8)	0.0025 (8)	−0.0034 (8)
C18	0.0445 (12)	0.0244 (10)	0.0322 (10)	−0.0104 (9)	−0.0014 (9)	0.0035 (8)
C19	0.0351 (11)	0.0249 (9)	0.0332 (10)	−0.0079 (8)	−0.0006 (8)	0.0012 (8)
C20	0.0311 (10)	0.0291 (10)	0.0340 (10)	−0.0044 (8)	−0.0008 (8)	0.0046 (8)
C21	0.0359 (11)	0.0282 (10)	0.0277 (9)	−0.0058 (8)	0.0035 (8)	0.0045 (8)
C22	0.0381 (12)	0.0342 (11)	0.0358 (11)	−0.0030 (9)	0.0027 (9)	0.0026 (9)
C23	0.0398 (12)	0.0524 (14)	0.0383 (12)	−0.0159 (11)	−0.0046 (10)	0.0085 (11)
C24	0.0742 (18)	0.0438 (13)	0.0240 (10)	−0.0242 (13)	0.0012 (11)	−0.0018 (9)
C25	0.0776 (19)	0.0396 (13)	0.0300 (11)	0.0034 (13)	0.0120 (11)	−0.0027 (10)
C26	0.0450 (13)	0.0441 (13)	0.0328 (11)	0.0052 (10)	0.0062 (9)	0.0029 (10)
S1B	0.0196 (3)	0.0276 (4)	0.0365 (5)	−0.0009 (2)	0.0041 (3)	−0.0067 (4)
O1B	0.0331 (10)	0.0299 (10)	0.0401 (10)	−0.0039 (8)	0.0021 (8)	−0.0060 (8)
O2B	0.0266 (12)	0.0406 (17)	0.0543 (16)	−0.0023 (12)	0.0052 (9)	−0.0097 (13)
O3B	0.0348 (10)	0.0405 (11)	0.0424 (11)	0.0038 (9)	−0.0033 (8)	−0.0013 (9)
C1B	0.0223 (16)	0.0286 (18)	0.039 (2)	0.0009 (11)	−0.0007 (16)	−0.0018 (17)
C2B	0.0306 (17)	0.0229 (15)	0.0367 (19)	0.0002 (15)	0.0111 (14)	−0.0021 (14)
C3B	0.0325 (18)	0.0293 (19)	0.034 (2)	0.0030 (16)	0.0089 (14)	0.0048 (16)
C4B	0.042 (2)	0.029 (2)	0.032 (2)	−0.0064 (13)	−0.0009 (17)	−0.0062 (17)
C5B	0.073 (3)	0.0317 (18)	0.068 (3)	0.019 (2)	−0.019 (2)	−0.0175 (18)
C6B	0.058 (3)	0.039 (2)	0.063 (3)	0.0226 (19)	−0.024 (2)	−0.016 (2)
C7B	0.056 (3)	0.036 (2)	0.0380 (19)	−0.0036 (19)	0.0014 (18)	−0.0039 (16)
S1A	0.0314 (3)	0.0229 (2)	0.0258 (2)	0.00075 (17)	−0.00353 (18)	0.00027 (17)
O1A	0.0562 (10)	0.0478 (10)	0.0265 (7)	−0.0112 (8)	0.0058 (7)	−0.0046 (7)
O2A	0.0397 (9)	0.0308 (8)	0.0600 (11)	0.0026 (7)	−0.0186 (8)	−0.0113 (7)
O3A	0.0590 (10)	0.0248 (7)	0.0296 (7)	0.0073 (7)	0.0030 (7)	0.0010 (6)
C1A	0.0259 (9)	0.0245 (9)	0.0280 (9)	0.0002 (7)	−0.0007 (7)	−0.0016 (7)
C2A	0.0352 (11)	0.0284 (10)	0.0290 (10)	−0.0043 (8)	−0.0015 (8)	0.0022 (8)
C3A	0.0355 (11)	0.0253 (10)	0.0412 (11)	−0.0051 (8)	−0.0029 (9)	−0.0014 (8)
C4A	0.0304 (10)	0.0373 (11)	0.0352 (11)	0.0000 (9)	−0.0021 (8)	−0.0079 (9)
C5A	0.0487 (13)	0.0400 (12)	0.0262 (10)	0.0022 (10)	−0.0023 (9)	0.0009 (9)
C6A	0.0449 (12)	0.0298 (10)	0.0296 (10)	0.0002 (9)	−0.0009 (9)	0.0034 (8)
C7A	0.0599 (16)	0.0458 (14)	0.0418 (13)	−0.0028 (12)	−0.0042 (11)	−0.0141 (11)
O1W	0.0231 (7)	0.0317 (8)	0.0483 (9)	0.0003 (6)	0.0047 (6)	−0.0126 (7)
O2W	0.0312 (9)	0.0375 (10)	0.0971 (17)	−0.0067 (8)	−0.0143 (10)	−0.0064 (10)

### Geometric parameters (Å, º)

**Table:**

N1—C4	1.500 (2)	C26—H26A	0.9500
N1—C1	1.504 (2)	S1B—O2B	1.446 (3)
N1—C5	1.532 (2)	S1B—O3B	1.454 (2)
N1—H1N	0.84 (3)	S1B—O1B	1.467 (2)
N2—C2	1.485 (2)	S1B—C1B	1.764 (5)
N2—C3	1.492 (2)	C1B—C6B	1.366 (5)
N2—C18	1.511 (3)	C1B—C2B	1.393 (6)
N2—H2N	0.98 (3)	C2B—C3B	1.390 (6)
C1—C2	1.521 (3)	C2B—H2BA	0.9500
C1—H1A	0.9900	C3B—C4B	1.400 (6)
C1—H1B	0.9900	C3B—H3BA	0.9500
C2—H2A	0.9900	C4B—C5B	1.368 (6)
C2—H2B	0.9900	C4B—C7B	1.527 (6)
C3—C4	1.511 (3)	C5B—C6B	1.392 (5)
C3—H3A	0.9900	C5B—H5BA	0.9500
C3—H3B	0.9900	C6B—H6BA	0.9500
C4—H4A	0.9900	C7B—H7BA	0.9800
C4—H4B	0.9900	C7B—H7BB	0.9800
C5—C6	1.518 (3)	C7B—H7BC	0.9800
C5—C12	1.520 (3)	S1C—O1C	1.432 (11)
C5—H5A	1.0000	S1C—O3C	1.478 (9)
C6—C7	1.385 (3)	S1C—O2C	1.515 (17)
C6—C11	1.395 (3)	S1C—C1C	1.751 (16)
C7—C8	1.384 (3)	C1C—C2C	1.33 (2)
C7—H7A	0.9500	C1C—C6C	1.44 (2)
C8—C9	1.391 (4)	C2C—C3C	1.354 (19)
C8—H8A	0.9500	C2C—H2CA	0.9500
C9—C10	1.378 (4)	C3C—C4C	1.36 (3)
C9—H9A	0.9500	C3C—H3CA	0.9500
C10—C11	1.384 (4)	C4C—C7C	1.44 (2)
C10—H10A	0.9500	C4C—C5C	1.50 (2)
C11—H11A	0.9500	C5C—C6C	1.385 (19)
C12—C17	1.385 (3)	C5C—H5CA	0.9500
C12—C13	1.392 (3)	C6C—H6CA	0.9500
C13—C14	1.390 (3)	C7C—H7CA	0.9800
C13—H13A	0.9500	C7C—H7CB	0.9800
C14—C15	1.384 (4)	C7C—H7CC	0.9800
C14—H14A	0.9500	S1A—O3A	1.4555 (15)
C15—C16	1.379 (4)	S1A—O1A	1.4564 (18)
C15—H15A	0.9500	S1A—O2A	1.4576 (16)
C16—C17	1.385 (3)	S1A—C1A	1.773 (2)
C16—H16A	0.9500	C1A—C6A	1.387 (3)
C17—H17A	0.9500	C1A—C2A	1.390 (3)
C18—C19	1.492 (3)	C2A—C3A	1.386 (3)
C18—H18A	0.9900	C2A—H2AA	0.9500
C18—H18B	0.9900	C3A—C4A	1.396 (3)
C19—C20	1.328 (3)	C3A—H3AA	0.9500
C19—H19A	0.9500	C4A—C5A	1.393 (3)
C20—C21	1.471 (3)	C4A—C7A	1.507 (3)
C20—H20A	0.9500	C5A—C6A	1.391 (3)
C21—C22	1.393 (3)	C5A—H5AA	0.9500
C21—C26	1.394 (3)	C6A—H6AA	0.9500
C22—C23	1.385 (3)	C7A—H7AA	0.9800
C22—H22A	0.9500	C7A—H7AB	0.9800
C23—C24	1.389 (4)	C7A—H7AC	0.9800
C23—H23A	0.9500	O1W—H1W1	0.84 (3)
C24—C25	1.372 (4)	O1W—H1W2	0.91 (4)
C24—H24A	0.9500	O2W—H2W1	0.86 (4)
C25—C26	1.375 (4)	O2W—H2W2	0.83 (5)
C25—H25A	0.9500		
			
C4—N1—C1	108.42 (14)	C22—C23—H23A	119.7
C4—N1—C5	109.94 (15)	C24—C23—H23A	119.7
C1—N1—C5	111.35 (14)	C25—C24—C23	119.2 (2)
C4—N1—H1N	109.8 (17)	C25—C24—H24A	120.4
C1—N1—H1N	104.4 (17)	C23—C24—H24A	120.4
C5—N1—H1N	112.7 (18)	C24—C25—C26	120.9 (2)
C2—N2—C3	109.37 (15)	C24—C25—H25A	119.6
C2—N2—C18	112.28 (16)	C26—C25—H25A	119.6
C3—N2—C18	110.54 (16)	C25—C26—C21	120.8 (2)
C2—N2—H2N	110.5 (16)	C25—C26—H26A	119.6
C3—N2—H2N	107.8 (16)	C21—C26—H26A	119.6
C18—N2—H2N	106.2 (17)	O2B—S1B—O3B	114.03 (19)
N1—C1—C2	110.92 (15)	O2B—S1B—O1B	110.68 (18)
N1—C1—H1A	109.5	O3B—S1B—O1B	112.54 (13)
C2—C1—H1A	109.5	O2B—S1B—C1B	106.23 (19)
N1—C1—H1B	109.5	O3B—S1B—C1B	107.02 (17)
C2—C1—H1B	109.5	O1B—S1B—C1B	105.72 (18)
H1A—C1—H1B	108.0	C6B—C1B—C2B	119.9 (4)
N2—C2—C1	111.45 (15)	C6B—C1B—S1B	120.1 (4)
N2—C2—H2A	109.3	C2B—C1B—S1B	119.9 (3)
C1—C2—H2A	109.3	C3B—C2B—C1B	119.8 (3)
N2—C2—H2B	109.3	C3B—C2B—H2BA	120.1
C1—C2—H2B	109.3	C1B—C2B—H2BA	120.1
H2A—C2—H2B	108.0	C2B—C3B—C4B	120.3 (4)
N2—C3—C4	111.13 (16)	C2B—C3B—H3BA	119.9
N2—C3—H3A	109.4	C4B—C3B—H3BA	119.9
C4—C3—H3A	109.4	C5B—C4B—C3B	118.6 (4)
N2—C3—H3B	109.4	C5B—C4B—C7B	120.6 (4)
C4—C3—H3B	109.4	C3B—C4B—C7B	120.7 (4)
H3A—C3—H3B	108.0	C4B—C5B—C6B	121.4 (4)
N1—C4—C3	111.03 (16)	C4B—C5B—H5BA	119.3
N1—C4—H4A	109.4	C6B—C5B—H5BA	119.3
C3—C4—H4A	109.4	C1B—C6B—C5B	120.0 (4)
N1—C4—H4B	109.4	C1B—C6B—H6BA	120.0
C3—C4—H4B	109.4	C5B—C6B—H6BA	120.0
H4A—C4—H4B	108.0	O1C—S1C—O3C	113.0 (6)
C6—C5—C12	113.64 (16)	O1C—S1C—O2C	112.5 (7)
C6—C5—N1	110.62 (15)	O3C—S1C—O2C	108.5 (8)
C12—C5—N1	111.31 (15)	O1C—S1C—C1C	107.6 (8)
C6—C5—H5A	107.0	O3C—S1C—C1C	108.4 (8)
C12—C5—H5A	107.0	O2C—S1C—C1C	106.6 (8)
N1—C5—H5A	107.0	C2C—C1C—C6C	119.8 (15)
C7—C6—C11	119.6 (2)	C2C—C1C—S1C	122.0 (15)
C7—C6—C5	122.63 (18)	C6C—C1C—S1C	118.2 (13)
C11—C6—C5	117.76 (19)	C1C—C2C—C3C	121.6 (16)
C8—C7—C6	119.8 (2)	C1C—C2C—H2CA	119.2
C8—C7—H7A	120.1	C3C—C2C—H2CA	119.2
C6—C7—H7A	120.1	C2C—C3C—C4C	126.2 (18)
C7—C8—C9	120.6 (2)	C2C—C3C—H3CA	116.9
C7—C8—H8A	119.7	C4C—C3C—H3CA	116.9
C9—C8—H8A	119.7	C3C—C4C—C7C	130.0 (18)
C10—C9—C8	119.6 (2)	C3C—C4C—C5C	112.0 (15)
C10—C9—H9A	120.2	C7C—C4C—C5C	118.0 (16)
C8—C9—H9A	120.2	C6C—C5C—C4C	122.2 (13)
C9—C10—C11	120.2 (2)	C6C—C5C—H5CA	118.9
C9—C10—H10A	119.9	C4C—C5C—H5CA	118.9
C11—C10—H10A	119.9	C5C—C6C—C1C	117.8 (13)
C10—C11—C6	120.2 (2)	C5C—C6C—H6CA	121.1
C10—C11—H11A	119.9	C1C—C6C—H6CA	121.1
C6—C11—H11A	119.9	C4C—C7C—H7CA	109.5
C17—C12—C13	118.55 (19)	C4C—C7C—H7CB	109.5
C17—C12—C5	116.74 (18)	H7CA—C7C—H7CB	109.5
C13—C12—C5	124.66 (18)	C4C—C7C—H7CC	109.5
C14—C13—C12	120.7 (2)	H7CA—C7C—H7CC	109.5
C14—C13—H13A	119.6	H7CB—C7C—H7CC	109.5
C12—C13—H13A	119.6	O3A—S1A—O1A	111.88 (11)
C15—C14—C13	119.9 (2)	O3A—S1A—O2A	113.10 (11)
C15—C14—H14A	120.1	O1A—S1A—O2A	111.82 (11)
C13—C14—H14A	120.1	O3A—S1A—C1A	106.39 (9)
C16—C15—C14	119.7 (2)	O1A—S1A—C1A	106.50 (10)
C16—C15—H15A	120.1	O2A—S1A—C1A	106.64 (10)
C14—C15—H15A	120.1	C6A—C1A—C2A	120.25 (19)
C15—C16—C17	120.3 (2)	C6A—C1A—S1A	121.34 (16)
C15—C16—H16A	119.8	C2A—C1A—S1A	118.41 (15)
C17—C16—H16A	119.8	C3A—C2A—C1A	119.8 (2)
C16—C17—C12	120.8 (2)	C3A—C2A—H2AA	120.1
C16—C17—H17A	119.6	C1A—C2A—H2AA	120.1
C12—C17—H17A	119.6	C2A—C3A—C4A	121.0 (2)
C19—C18—N2	112.09 (18)	C2A—C3A—H3AA	119.5
C19—C18—H18A	109.2	C4A—C3A—H3AA	119.5
N2—C18—H18A	109.2	C5A—C4A—C3A	118.4 (2)
C19—C18—H18B	109.2	C5A—C4A—C7A	120.8 (2)
N2—C18—H18B	109.2	C3A—C4A—C7A	120.8 (2)
H18A—C18—H18B	107.9	C6A—C5A—C4A	121.2 (2)
C20—C19—C18	122.1 (2)	C6A—C5A—H5AA	119.4
C20—C19—H19A	118.9	C4A—C5A—H5AA	119.4
C18—C19—H19A	118.9	C1A—C6A—C5A	119.5 (2)
C19—C20—C21	126.3 (2)	C1A—C6A—H6AA	120.3
C19—C20—H20A	116.8	C5A—C6A—H6AA	120.3
C21—C20—H20A	116.8	C4A—C7A—H7AA	109.5
C22—C21—C26	118.4 (2)	C4A—C7A—H7AB	109.5
C22—C21—C20	123.0 (2)	H7AA—C7A—H7AB	109.5
C26—C21—C20	118.6 (2)	C4A—C7A—H7AC	109.5
C23—C22—C21	120.2 (2)	H7AA—C7A—H7AC	109.5
C23—C22—H22A	119.9	H7AB—C7A—H7AC	109.5
C21—C22—H22A	119.9	H1W1—O1W—H1W2	98 (3)
C22—C23—C24	120.5 (2)	H2W1—O2W—H2W2	114 (4)
			
C4—N1—C1—C2	57.1 (2)	C22—C21—C26—C25	0.7 (3)
C5—N1—C1—C2	178.22 (15)	C20—C21—C26—C25	−177.7 (2)
C3—N2—C2—C1	56.7 (2)	O2B—S1B—C1B—C6B	−111.7 (4)
C18—N2—C2—C1	179.75 (16)	O3B—S1B—C1B—C6B	10.5 (5)
N1—C1—C2—N2	−57.9 (2)	O1B—S1B—C1B—C6B	130.7 (4)
C2—N2—C3—C4	−57.2 (2)	O2B—S1B—C1B—C2B	64.8 (4)
C18—N2—C3—C4	178.65 (17)	O3B—S1B—C1B—C2B	−173.0 (3)
C1—N1—C4—C3	−58.0 (2)	O1B—S1B—C1B—C2B	−52.8 (4)
C5—N1—C4—C3	−179.91 (15)	C6B—C1B—C2B—C3B	1.1 (7)
N2—C3—C4—N1	59.2 (2)	S1B—C1B—C2B—C3B	−175.5 (3)
C4—N1—C5—C6	172.46 (15)	C1B—C2B—C3B—C4B	−1.3 (6)
C1—N1—C5—C6	52.3 (2)	C2B—C3B—C4B—C5B	1.1 (7)
C4—N1—C5—C12	−60.2 (2)	C2B—C3B—C4B—C7B	178.2 (4)
C1—N1—C5—C12	179.62 (15)	C3B—C4B—C5B—C6B	−0.6 (8)
C12—C5—C6—C7	−49.0 (2)	C7B—C4B—C5B—C6B	−177.8 (5)
N1—C5—C6—C7	77.0 (2)	C2B—C1B—C6B—C5B	−0.6 (8)
C12—C5—C6—C11	130.33 (19)	S1B—C1B—C6B—C5B	175.9 (4)
N1—C5—C6—C11	−103.6 (2)	C4B—C5B—C6B—C1B	0.4 (9)
C11—C6—C7—C8	−0.2 (3)	O1C—S1C—C1C—C2C	−37.5 (16)
C5—C6—C7—C8	179.20 (19)	O3C—S1C—C1C—C2C	−160.0 (13)
C6—C7—C8—C9	−0.2 (3)	O2C—S1C—C1C—C2C	83.4 (15)
C7—C8—C9—C10	0.2 (4)	O1C—S1C—C1C—C6C	141.7 (11)
C8—C9—C10—C11	0.1 (4)	O3C—S1C—C1C—C6C	19.2 (14)
C9—C10—C11—C6	−0.5 (4)	O2C—S1C—C1C—C6C	−97.4 (13)
C7—C6—C11—C10	0.5 (3)	C6C—C1C—C2C—C3C	2 (2)
C5—C6—C11—C10	−178.9 (2)	S1C—C1C—C2C—C3C	−178.6 (13)
C6—C5—C12—C17	−94.7 (2)	C1C—C2C—C3C—C4C	1 (3)
N1—C5—C12—C17	139.64 (18)	C2C—C3C—C4C—C7C	178.0 (19)
C6—C5—C12—C13	82.6 (3)	C2C—C3C—C4C—C5C	−4 (3)
N1—C5—C12—C13	−43.1 (3)	C3C—C4C—C5C—C6C	6 (2)
C17—C12—C13—C14	0.8 (4)	C7C—C4C—C5C—C6C	−176.3 (15)
C5—C12—C13—C14	−176.4 (2)	C4C—C5C—C6C—C1C	−3.6 (19)
C12—C13—C14—C15	0.6 (4)	C2C—C1C—C6C—C5C	−1 (2)
C13—C14—C15—C16	−1.0 (4)	S1C—C1C—C6C—C5C	−179.8 (10)
C14—C15—C16—C17	−0.1 (4)	O3A—S1A—C1A—C6A	−11.5 (2)
C15—C16—C17—C12	1.5 (3)	O1A—S1A—C1A—C6A	−131.02 (19)
C13—C12—C17—C16	−1.9 (3)	O2A—S1A—C1A—C6A	109.44 (19)
C5—C12—C17—C16	175.6 (2)	O3A—S1A—C1A—C2A	169.05 (17)
C2—N2—C18—C19	63.1 (2)	O1A—S1A—C1A—C2A	49.6 (2)
C3—N2—C18—C19	−174.45 (18)	O2A—S1A—C1A—C2A	−69.98 (19)
N2—C18—C19—C20	−124.4 (2)	C6A—C1A—C2A—C3A	−1.0 (3)
C18—C19—C20—C21	−172.35 (19)	S1A—C1A—C2A—C3A	178.44 (17)
C19—C20—C21—C22	−13.5 (3)	C1A—C2A—C3A—C4A	0.9 (3)
C19—C20—C21—C26	164.8 (2)	C2A—C3A—C4A—C5A	−0.1 (3)
C26—C21—C22—C23	0.1 (3)	C2A—C3A—C4A—C7A	−179.2 (2)
C20—C21—C22—C23	178.4 (2)	C3A—C4A—C5A—C6A	−0.6 (4)
C21—C22—C23—C24	−0.9 (4)	C7A—C4A—C5A—C6A	178.6 (2)
C22—C23—C24—C25	0.8 (4)	C2A—C1A—C6A—C5A	0.3 (3)
C23—C24—C25—C26	0.0 (4)	S1A—C1A—C6A—C5A	−179.06 (18)
C24—C25—C26—C21	−0.8 (4)	C4A—C5A—C6A—C1A	0.4 (4)

### Hydrogen-bond geometry (Å, º)

**Table:**

*D*—H···*A*	*D*—H	H···*A*	*D*···*A*	*D*—H···*A*
N1—H1*N*···O1*W*	0.84 (3)	1.86 (3)	2.699 (2)	174 (3)
N2—H2*N*···O2*W*	0.98 (3)	1.69 (3)	2.661 (3)	173 (3)
O1*W*—H1*W*1···O1*B*^i^	0.84 (3)	1.86 (3)	2.685 (2)	167 (3)
O1*W*—H1*W*1···O3*C*^i^	0.84 (3)	2.07 (3)	2.826 (9)	149 (3)
O1*W*—H1*W*2···O3*A*	0.91 (4)	1.85 (4)	2.747 (2)	168 (4)
O2*W*—H2*W*1···O2*A*^ii^	0.86 (4)	1.88 (4)	2.739 (3)	176 (3)
O2*W*—H2*W*2···O1*C*	0.83 (5)	1.59 (5)	2.336 (10)	147 (4)
O2*W*—H2*W*2···O1*B*	0.83 (5)	2.01 (5)	2.830 (3)	169 (4)

Symmetry codes: (i) *x*−1, *y*, *z*; (ii) *x*+1, *y*, *z*.
